# Expanding the Roles of Medical Educators in the Generative Artificial Intelligence Era: A Qualitative Study

**DOI:** 10.1007/s40670-026-02734-3

**Published:** 2026-04-28

**Authors:** Ikuo Shimizu, Hajime Kasai, Naoto Ozaki, Hiromu Nishiuchi, Kiyoshi Shikino, Nobuyuki Araki, Misaki Onodera, Yasuhiko Kimura, Tomoko Tsukamoto, Hiroshi Tajima, Shoichi Ito, Eiryo Kawakami

**Affiliations:** 1 Department of Medical Education, Graduate School of Medicine, Chiba University, 1-8-1 Inohana, Chiba, 2608672 Japan; 2 Data Science Core, Chiba University, Chiba, Japan; 3 Department of Community-Oriented Medical Education, Graduate School of Medicine, Chiba University, Chiba, Japan; 4 Health Professional Development Center, Chiba University Hospital, Chiba, Japan; 5 Department of Artificial Intelligence Medicine, Graduate School of Medicine, Chiba University, Chiba, Japan

**Keywords:** Generative artificial intelligence, Medical education, Medical educator, Digital transformation, Qualitative research

## Abstract

The rapid advancement of generative artificial intelligence (GAI) necessitates reconceptualizing medical educators’ professional roles. While Harden’s Six Roles of the Teacher have long defined educator identity, GAI integration may require expansion beyond this established framework. This study explored how medical educators perceive role evolution in the GAI era and identified emerging educator functions. A qualitative descriptive study was conducted during a faculty development program at a Japanese medical school in August 2025. Fifty-two faculty members discussed improving education using GAI. From 137 analyzed statements, content analysis applied Harden’s framework deductively, while the SAMR model assessed transformation levels. Statements showing high transformation (Modification/Redefinition) underwent inductive analysis to identify new roles. Traditional roles dominated: resource developer (*n* = 34), assessor (*n* = 29), facilitator (*n* = 29), information provider (*n* = 23), planner (*n* = 17), and role model (*n* = 5). Most statements reflected enhancement of existing practices (Substitution *n* = 51; Augmentation *n* = 67). Critically, 19 statements demonstrated transformative potential (Modification *n* = 15; Redefinition *n* = 4), revealing five emergent educator roles: analyst (evaluating GAI outputs), coordinator (orchestrating human-AI collaboration), content generator (co-creating with AI), fact checker (verifying AI accuracy), and lifelong learner (continuously adapting to AI evolution). This study demonstrates that GAI is expanding medical educators’ professional identity beyond traditional frameworks. While GAI enhances conventional roles, it simultaneously creates new responsibilities requiring critical judgment, ethical oversight, and adaptive expertise. These findings challenge the notion of AI as mere replacement, instead positioning educators as essential mediators in human-AI educational ecosystems. Faculty development must incorporate AI literacy and reflective practice to support this expanded professional identity.

## Background

In recent years, generative artificial intelligence (GAI) has emerged as one of the most transformative technologies in education and healthcare. Large language models (LLMs) have rapidly evolved in their ability to generate human-like text, synthesize information, and support reasoning tasks across a wide range of professional domains [1]. Within the medical field, these tools are increasingly used to assist clinical documentation, diagnostic reasoning, literature review, and patient communication. In parallel, their influence extends to health professions’ education, reshaping how students learn, educators teach, and institutions define competence in the digital era [2]. For instance, a recent scoping review found that GAI is already being explored for self-directed learning, simulation scenarios, and writing assistance in medical education [3].

GAI has demonstrated substantial potential for medical education. It can simulate clinical scenarios, generate personalized feedback, summarize complex scientific evidence, and support multilingual communication in global learning environments [4–6]. For example, several studies indicate that LLMs can provide immediate formative feedback on students’ reflective writing or problem-based learning reports with a level of consistency that rivals human tutors [7]. In clinical-reasoning education, GAI tools have been applied to generate differential diagnoses or critique student diagnostic processes, thereby promoting metacognitive awareness. Moreover, GAI-assisted simulation designs allow educators to create patient cases or objective structured clinical examination checklists more efficiently, enhancing the scalability of training in communication and patient safety [8, 9]. However, the widespread adoption of these tools also raises questions about the evolving role and identity of medical educators. The exponential pace of GAI development has created an increasing gap between technological capabilities and established educational frameworks. This rapid evolution presents an urgent challenge for educators struggling to keep pace, necessitating a fundamental paradigm shift in how professional roles are conceptualized.

Traditionally, the role of the medical teacher has been conceptualized through frameworks such as Harden’s “Six Roles of the Teacher” in medical education [10]. Harden classified the teacher’s functions into six broad roles: information provider (in lectures and clinical settings), role model (on-the-job and as a professional), facilitator (of learning and reflection), assessor (of competence and performance), planner (of curricula and courses), and resource developer (of educational materials). This framework was developed in an era when the primary sources of information were textbooks and teachers themselves, and when the educator’s expertise was indispensable for guiding learners through a relatively fixed curriculum. However, the accelerating integration of GAI into both educational and clinical systems is beginning to challenge these traditional boundaries.

The widespread availability of GAI tools capable of generating explanations, case analyses, and even personalized learning materials challenges the conventional assumption that teachers serve as the primary source of information and guidance [11, 12]. Students and clinicians alike can now query GAI systems to obtain immediate, context-specific insights that were once accessible only through human experts. These systems are already being applied in diverse educational and clinical contexts—such as generating clinical case scenarios for simulation training, drafting patient discharge summaries, supporting literature searches in evidence-based medicine courses, or producing multilingual communication scripts for standardised patient encounters [13–15]. In diagnostic education, GAI tools have been used to provide structured feedback on students’ reasoning processes or to simulate differential diagnoses based on patient data [16, 17]. In research and publication training, GAI assists in refining abstracts, producing graphical summaries, and improving linguistic clarity for non-native English speakers [18].

If GAI can perform some cognitive and communicative tasks traditionally undertaken by educators, how can educational programs ensure that learners develop critical appraisal skills rather than an unreflective dependence on algorithmic output? Additionally, how should institutions support teachers in navigating these technological shifts while maintaining ethical and educational standards? These questions underscore the need to reconsider the established frameworks of medical educators considering the ongoing digital transformation.

Rather than assuming that GAI will simply replace certain aspects of teaching, it may be more appropriate to view this as a moment of digital transformation—one that requires educators to redefine their professional identity and responsibilities in the context of human–GAI collaboration. To respond effectively, educators must engage in reflective practice, develop digital and ethical literacy, and re-examine how they contribute to cultivating trustworthy, empathetic, and adaptive professionals in an GAI-augmented healthcare system [19].

Against this backdrop, this study explores the central question: What are the roles required of medical educators in the era of GAI? This work aims to clarify how GAI is reshaping traditional educational paradigms and to propose directions for redefining the professional identity of educators in the coming decades.

## Methods

### Study Design and Context

This study employed a qualitative descriptive design, utilizing conventional content analysis [20]. It was conducted as part of a faculty development program at a medical school in Japan in August 2025. The retreat program is an annual institutional initiative that promotes curriculum development and pedagogical innovation among faculty members. Before the group discussions, all the participants engaged in hands-on exercises with generative AI applications, which created a shared baseline of understanding and enabled a more balanced dialogue. This study followed the Standards for Reporting Qualitative Research [21].

### Participants

Fifty-two faculty members affiliated with the medical school and university hospital participated in the study. All participants were licensed physicians. They represented a range of academic ranks (12 professors, 21 associate professors, eight lecturers, 10 assistant professors, and one other) and diverse educational fields, including 27 members from clinical departments (e.g., internal medicine, surgery, and psychiatry) and 25 members from basic science departments (e.g., physiology, pharmacology, and public health). Most participants reported having heard of generative AI but had limited prior experience with its regular use. Participation was voluntary and open to all program attendees.

Prior to the workshop, participants were provided with a digital information document and received a verbal briefing outlining the study’s objectives and data handling protocols. This study utilized an implied consent model, in which the voluntary completion and submission of the surveys were considered as providing consent for the data to be used for research purposes. To ensure a clear opt-out mechanism, participants were explicitly informed that they could decline participation at any time by requesting that their responses be excluded from the study.

### Data Collection

Data were collected during the group-work component of the workshop, which explored the question: “What can medical educators do to improve education using GAI?”

Following introductory lectures and hands-on sessions on GAI, the participants were divided into small groups of four to five members. Each group discussed the potential educational applications of GAI and recorded their ideas on online sticky notes at Google Spreadsheet. These notes were collected, anonymized, and transcribed into textual data. Each statement was treated as an analytical unit, representing the faculty members’ perceptions of how GAI could influence teaching and learning.

### Data Analysis

Qualitative content analysis was conducted by three researchers (IS, HK, NO), who independently coded all the statements. The analysis combined deductive and inductive approaches: statements corresponding to Harden’s Six Roles of the Teacher (information provider, role model, facilitator, assessor, planner, and resource developer) were coded deductively, whereas the remaining statements were analyzed inductively to identify emerging themes.

After coding, the influence of GAI on each statement was evaluated using the Substitution-Augmentation-Modification-Redefinition (SAMR) model, which describes how technology impacts learning [22, 23]. This model enabled researchers to understand how participants perceived the integration of AI into educational practice, ranging from the incremental substitution of existing teaching tasks to the fundamental redefinition of educators’ roles. Each coded statement was assigned to one of four SAMR levels according to the implied depth of transformation. Statements that reached the Modification or Redefinition levels were interpreted as reflecting novel educator roles arising from digital transformation and were grouped into new thematic categories beyond Harden’s traditional framework [24].

Multiple strategies were applied throughout the study to enhance the validity and trustworthiness of the analyses. Each coder first conducted independent coding, and the results were compared and refined through consensus meetings to ensure analytical triangulation. All coding decisions and revisions were documented in a shared digital codebook to maintain an audit trail. The preliminary category structure was reviewed by two external experts in medical education who were not involved in data collection and served as peer debriefers to ensure confirmability. As a member check, a subset of workshop participants reviewed the synthesized findings to verify their consistency with the original discussion context. Reflexive memos were maintained to record researchers’ perspectives and potential biases, contributing to transparency and interpretive rigor.

All data were originally written and analyzed in Japanese. Coding and category generation were conducted in the source language to preserve the contextual and cultural nuances. Representative excerpts and category labels were translated into English for reporting. Based on the emergent categories and subsequent thematic synthesis through iterative discussions among the authors, a final thematic framework was developed. This framework integrated categories aligned with Harden’s six roles as well as new roles associated with digital transformation in the era of GAI.

The research team comprised medical educators with professional experience in qualitative research, faculty development, and patient safety education. Their backgrounds informed, but did not predetermine, data interpretation. The analytical process emphasized credibility through triangulation and member checking, dependability through the audit trail, and confirmability through external peer review.

### Ethical Considerations

This study was approved by the Ethics Committee of the Graduate School of Medicine, Chiba University (Approval No. 3425). Participation was voluntary, and all collected data were anonymized before analysis. The ethical procedures were identical to those used in previous studies on GAI in medical education.

## Results

A total of 141 textual statements were collected from the faculty group discussions. Four statements unrelated to the research question, such as comments on administrative tasks, were excluded. The remaining 137 statements were analyzed as individual units of meaning and coded according to Harden’s six roles of the medical teacher. The distribution of the coded statements was as follows: resource developer (*n* = 34, 24.8%), assessor (*n* = 29, 21.2%), facilitator (*n* = 29, 21.2%), information provider (*n* = 23, 16.8%), planner (*n* = 17, 12.4%), and role model (*n* = 5, 3.6%). Most faculty members’ comments covered a wide range of perspectives on the use of GAI within these traditional roles.

The faculty members’ comments covered a wide range of perspectives on the use of GAI within the traditional roles of the medical educator.

The assessor role included statements describing the potential for GAI to support formative assessment, create or refine rubrics, and generate individualized feedback for students. The planner category encompassed opinions on integrating GAI into curriculum design, organizing learning sequences, and developing educational policies. In the resource developer category, participants mentioned creating instructional materials, simulation cases, and teaching aids with the help of GAI.

Statements assigned to the role of the information provider described using GAI for literature searches, summarizing medical content, and providing supplementary explanations to students. The facilitator category reflected the view that GAI could help teachers provide learning support and individualized guidance. Finally, statements under the role model category emphasized the importance of demonstrating the ethical, professional, and responsible use of GAI tools in education and clinical training.

### Roles Reflecting Digital Transformation

After the initial coding based on Harden’s framework, each statement was further classified using the SAMR model to determine the extent of technology implementation implied in the faculty members’ views. Most statements were categorized at the Substitution (*n* = 51) or Augmentation (*n* = 67) levels, indicating that many participants perceived GAI primarily as an enhancement of existing educational practices. Nineteen statements were classified as Modification (*n* = 15) or Redefinition (*n* = 4), representing a higher level of transformation of the educator’s function (Table 1).

**Table 1:** Distribution of Statements Across Harden’s Six Roles

	Role, *n* (%)	Substitution, *n* (%)	Augmentation, *n* (%)	Modification, *n* (%)	Redefinition, *n* (%)
Resource developer	34 (24.8)	10 (19.6)	15 (22.4)	5 (33.3)	4 (100)
Assessor	29 (21.2)	13 (25.5)	16 (23.9)	0 (0)	0 (0)
Facilitator	29 (21.2)	19 (37.3)	9 (13.4)	1 (6.7)	0 (0)
Information provider	23 (16.8)	3 (5.9)	17 (25.4)	3 (20.0)	0 (0)
Planner	17 (12.4)	4 (7.8)	7 (10.4)	6 (40.0)	0 (0)
Role model	5 (3.6)	2 (3.9)	3 (4.5)	0 (0)	0 (0)
Total	137 (100)	51 (37.2)	67 (49.0)	15 (10.9)	4 (2.9)

Following this classification, these 19 statements were reanalyzed inductively to identify new thematic categories representing emerging educator roles in the context of digital transformation (Table 2). Five new roles were identified:

- **Coordinator (*n*** = **6)**: Teachers who manage collaboration between humans and GAI systems. For instance, one participant suggested “having GAI deliver the lecture while the instructor conducts a flipped-classroom session,” illustrating a shift toward educational orchestration.
- **Content Generator (*n*** = **4)**: Teachers who utilize GAI to create novel instructional goals. A representative proposal included “conducting GAI-based role-play of patient interviews prior to clinical rotations,” which enables simulation at a scale previously difficult to achieve.
- **Analyst (*n*** = **4)**: Teachers who use GAI to monitor learning processes. Participants envisioned “incorporating GAI tools into preparation for the national licensing examination” to draw insights for educational improvement.
- **Lifelong Learner (*n*** = **3)**: Teachers who adapt their professional roles by “applying GAI to proofread and refine lecture content,” serving as role models for technological adaptation.
- **Fact Checker (*n*** = **2)**: Teachers who maintain integrity by “engaging GAI tools to maintain up-to-date clinical and educational knowledge” while verifying the accuracy of AI-generated information.

**Fig. 1:**
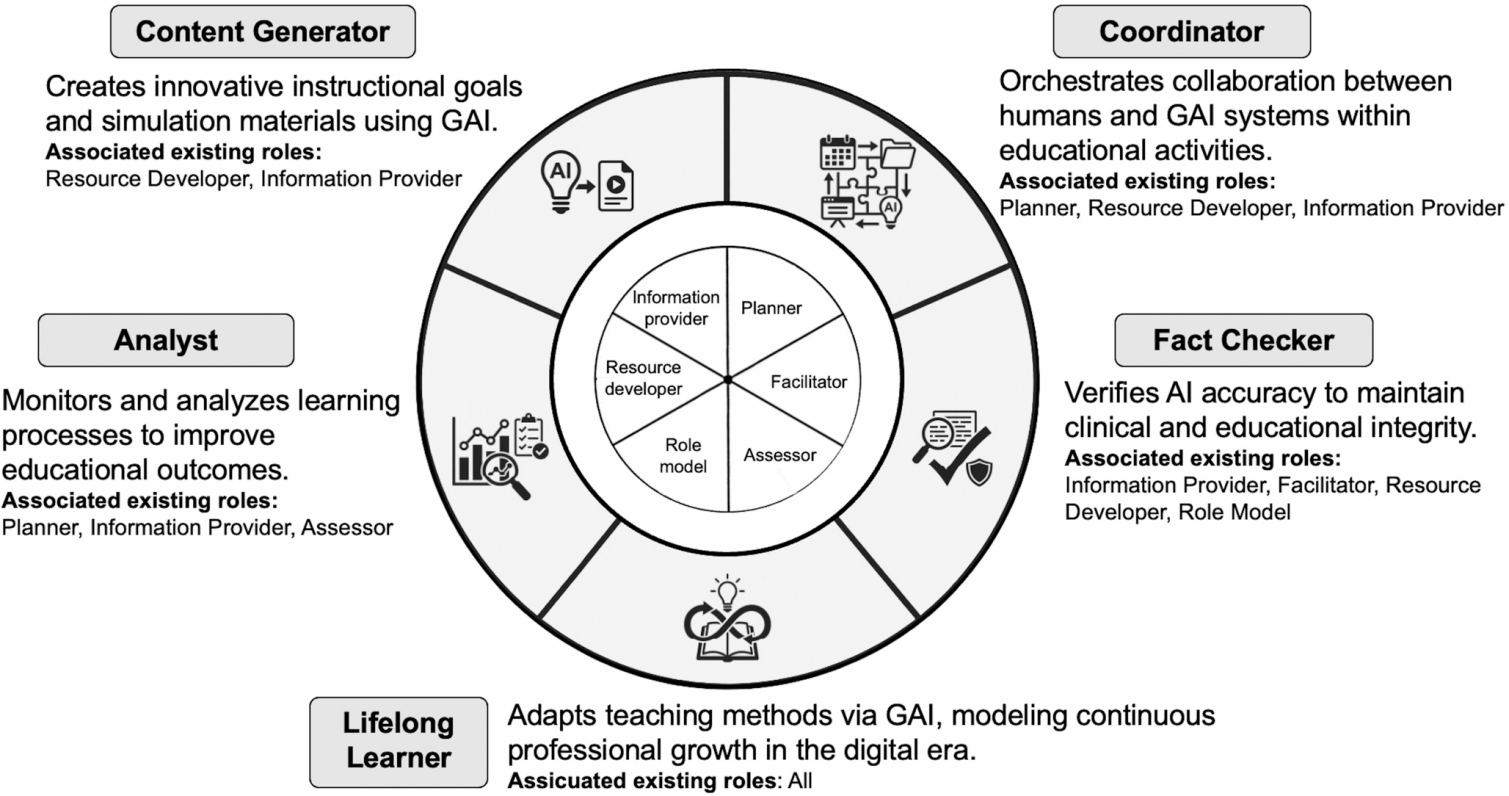
Conceptual Framework of Evolving Medical Educator Roles in the GAI Era

Representative quotations for each emergent role are presented in Table 2, illustrating how faculty members describe these new functions in the context of GAI. The conceptual integration of these emergent roles within Harden’s traditional framework is synthesized in Fig. 1.

**Table 2:** Emergent educator roles derived from Modification- and Redefinition- level statements, with their distribution across Harden’s six roles and representative examples

	*n*	RD	A	F	IP	*P*	RM	Representative quotes
Coordinator	6	2				4		Having GAI deliver the lecture while the instructor conducts a flipped-classroom session(RD -Modification)
								Allowing GAI to propose ideas for vertical integration between basic and clinical sciences(P-Modification)
Content generator	4	4						Conducting GAI-based role-play of patient interviews prior to clinical rotations(RD-Redefinition)
								Consulting GAI to identify anticipated patient questions in advance and preparing explanations accordingly(RD-Modification)
Analyst	4				2	2		Incorporating GAI tools into preparation for the national licensing examination(IP -Modification)
Lifelong learner	3	2			1			Applying GAI to proofread and refine lecture content(RD-Modification)
Fact checker	2			1	1			Engaging GAI tools to maintain up-to-date clinical and educational knowledge (IP-Modification)

RD = Resource developer; A = Assessor; F = Facilitator; IP = Information provider; P = Planner; RM = Role model

This framework illustrates the integration of five emergent roles identified in this study within the established paradigm of medical education. The thematic structure was synthesized through a collaborative consensus process among the authors, mapping the emergent categories onto Harden’s six existing role areas (represented by the inner segments). The outer layers represent the functional intersections of the emergent roles—Coordinator, Content Generator, Analyst, Lifelong Learner, and Fact Checker—with their associated existing roles.

## Discussion

This study examined how medical educators perceive their professional roles in the era of GAI by analyzing qualitative data collected from faculty members within a structured faculty development program. Using Harden’s Six Roles of the Teacher as a conceptual lens and the SAMR model to assess the degree of digital transformation, 137 faculty statements were categorized into six traditional educator roles. Asubset of statements revealed emerging roles beyond the previous framework. These findings indicate that medical educators primarily viewed GAI as a supportive tool for existing educational functions but also recognized new responsibilities related to analysis, creation, coordination, verification, and continuous learning in digitally enhanced learning environments.

The present findings demonstrate that GAI can substitute for and augment traditional educational functions, particularly resource development, knowledge delivery, assessment, and facilitation. Faculty members often describe using GAI to create simulation cases, generate formative feedback, and search for or summarize medical information, which is consistent with previous literature that identifies GAI as a tool for efficiency and personalization rather than replacement [25, 26]. These applications enhance productivity but do not diminish the educator’s central role in ensuring accuracy, context relevance, and ethical oversight. For instance, in assessment and facilitation, participants anticipated that GAI could streamline feedback processes and tailor learning experiences, while the educator retained responsibility for professional judgment and decision-making. Thus, the educators’ expertise evolves from information transmission toward the orchestration of human–GAI collaboration, a transition consistent with recent frameworks emphasizing educational content knowledge in the digital age [27].

Furthermore, the integration of the SAMR model into the analysis highlighted the areas in which GAI prompted a deeper transformation of educational functions. Analysis of statements at the modification level identified several new roles: analyst, coordinator, content generator, fact checker, and lifelong learner. The analyst role captures educators’ use of GAI-mediated data to interpret learning patterns and optimize instructional decisions, whereas the coordinator role involves managing the interface between human and machine contributions in educational workflows. The fact checker underscores the responsibility to verify the integrity and accuracy of AI-generated content, a function increasingly discussed under the broader concept of digital professionalism [28], which emphasizes ethical judgment, transparency, and responsible data use in technology-enhanced education.

In addition to these evaluative and managerial functions, the content generator role reflects a qualitatively distinct dimension of digital transformation. While Harden’s original resource developer role focuses on designing, organizing, and adapting educational materials based on existing curricular structures, the content generator role involves creating entirely new instructional resources or learning experiences—such as GAI-generated simulation scenarios or innovative proposals for vertical integration across basic and clinical domains. These activities illustrate how GAI can augment educators’ creative capacity and enable instructional innovations that were previously limited by time, expertise, or cognitive load.

Building on these evolving responsibilities, the lifelong learner role highlights the expectation that educators continuously update their competencies to sustain digital professionalism and effectively navigate GAI-enhanced educational environments. Simultaneously, the limited emergence of Redefinition-level roles underscores the ongoing challenges faced by institutional and curricular constraints in undergraduate medical education.

These findings suggest that generative AI will not diminish the importance of teachers but will require them to exercise higher levels of judgment, ethical awareness, and educational flexibility. Therefore, faculty development programs should incorporate experiential training and AI literacy education to enable teachers to engage confidently and responsibly with emerging technologies. The participants in this study represented a diverse range of positions and digital literacy levels. Such experiential learning is recognized as a key condition for meaningful faculty development when introducing new educational technologies [29, 30]. Providing educators with guided opportunities to explore AI functions helps to reduce anxiety regarding misinformation, bias, and ethical risks, which are frequently cited as barriers to AI adoption in higher education [31]. In this context, structured AI training and literacy programs may not only foster confidence but also promote responsible and informed use of generative technologies in academic and clinical education. For medical schools, building shared competence among faculty could serve as a foundation for developing institution-wide policies for safe and pedagogically sound AI utilization.

A notable result of this study was the relatively small number of the statements coded under the role model function. This suggests that, despite the expanding potential of GAI in instructional activities, participants did not perceive GAI as substantially contributing to the modeling of professional attitudes and behaviors. This lack of emphasis may stem from a perception among faculty that professional identity formation is fundamentally rooted in human presence, interpersonal vulnerability, and the shared emotional burden of clinical care—qualities perceived as difficult for AI to replicate. This finding corresponds with the importance of learning aside from GAI [32], which emphasizes that certain aspects of professional identity formation require direct human exemplars. Furthermore, because the paradigm of learning with GAI [32] is still in its infancy, established standards for modeling professional behaviors in an GAI-augmented environment remain undefined. However, as digital transformation progresses, there will be an increasing need for educators who can embody digital professionalism—demonstrating the ethical, critical, and transparent use of GAI as a new dimension of the health professionals [33]. Accordingly, even as GAI supports various educational roles, deliberate opportunities for human-to-human role modeling should remain integral within medical education.

This study had several limitations. First, this study conducted at a single Japanese medical school, and cultural or institutional characteristics may have influenced participants’ perspectives. Educational systems and professional hierarchies differ across countries, and these contextual factors may shape how educators conceptualize GAI integration. Second, the participant group primarily consisted of physicians involved in undergraduate education, with limited representation from other health professions, such as nursing or pharmacy. Consequently, the identified roles may not fully reflect interprofessional perspectives. Third, the participants’ familiarity with GAI varied widely, and some responses were based on hypothetical assumptions rather than on sustained practical experiences. Future research should therefore explore how educators’ perceptions evolve as GAI becomes embedded in everyday teaching practice and as faculty literacy levels increase through systematic training.

## Conclusion

This study explored how GAI is reshaping the professional identity of medical educators by examining faculty perceptions within the framework of Harden’s Six Roles of the Teacher and the SAMR model. Most faculty members viewed GAI as a tool to enhance existing teaching practices—particularly in assessment, facilitation, and resource development—rather than as a replacement for human educators. However, through digital transformation, new educator roles emerged: analyst, coordinator, content generator, fact checker, and lifelong learner. These roles highlight the expanding need for educators to manage GAI-driven processes, ensure the validity of generated content, and continuously adapt to technological innovations. Accordingly, institutions should prioritize targeted faculty development for these emergent roles, focusing especially on the coordinator and fact checker as urgent competencies to ensure educational integrity and patient safety.

Future research should investigate how these newly identified roles evolve through the longitudinal implementation of GAI-integrated curricula and how they influence learner outcomes, professionalism, and ethical reasoning. Ultimately, the goal is not to automate teaching but to empower educators to navigate the evolving intersection of human expertise and artificial intelligence in the pursuit of safer and more effective medical education.

## Data Availability

Data is stored in the Chiba University Repository for Access To Outcomes from Research (https://opac.ll.chiba-u.jp/da/curator/) and is available on request.
